# Vitamin C deficiency causes muscle atrophy and a deterioration in physical performance

**DOI:** 10.1038/s41598-019-41229-7

**Published:** 2019-03-20

**Authors:** Shoko Takisawa, Tomoko Funakoshi, Tomofumi Yatsu, Kisaburo Nagata, Toshiro Aigaki, Shuichi Machida, Akihito Ishigami

**Affiliations:** 1 Molecular Regulation of Aging, Tokyo Metropolitan Institute of Gerontology, Tokyo, 173-0015 Japan; 2 Cellular Genetics, Graduate School of Science and Engineering, Tokyo Metropolitan University, Tokyo, 192-0397 Japan; 3 Department of Biomolecular Science, Faculty of Science, Toho University, Chiba, 274-8510 Japan; 4 Graduate School of Health and Sports Science, Juntendo University, Chiba, 270-1695 Japan

## Abstract

L-Ascorbic acid (AsA) is a water-soluble antioxidant. We examined the effect of AsA deficiency on skeletal muscle using senescence marker protein-30 (SMP30)-knockout (KO) mice that are defective in AsA biosynthesis, which makes this mouse model similar to humans, to clarify the function of AsA in skeletal muscle. Eight-week-old female SMP30-KO mice were divided into the following two groups: an AsA-sufficient group [AsA(+)] that was administered 1.5 g/L AsA and an AsA-deficient group [AsA(−)] that was administered tap (AsA-free) water. At 4 weeks, the AsA content in the gastrocnemius muscle of AsA(−) mice was 0.7% compared to that in the gastrocnemius muscle of AsA(+) mice. Significantly lower weights of all muscles were observed in AsA(−) mice than those in AsA(+) mice at 12 and 16 weeks. The cross-sectional area of the soleus was significantly smaller in AsA(−) mice at 16 weeks than that in AsA(+) mice. The physical performance of AsA(−) mice was significantly less than that of AsA(+) mice at 12 weeks. Following AsA deficiency for 12 weeks, the expression of ubiquitin ligases, such as atrogin1/muscle atrophy F-box (MAFbx) and muscle RING-finger protein 1 (MuRF1), was upregulated. Furthermore, all detected effects of AsA deficiency on muscles of the AsA(−) group at 12 weeks were restored following AsA supplementation for 12 weeks. Thus, longer-term AsA deficiency is associated with muscle wasting, that this can be reversed by restoring AsA levels.

## Introduction

L-Ascorbic acid (AsA, vitamin C) is a water-soluble antioxidant that scavenges reactive oxygen species (ROS), such as hydroxyl radicals, singlet oxygen, and superoxide radicals^1^. Many vertebrates have the ability to synthesize AsA^2,3^. However, primates, including humans, and guinea pigs are unable to synthesize AsA since these organisms carry multiple mutations in the *Gulo* gene encoding L-gulono-γ-lactone oxidase, the last enzyme in the AsA biosynthesis pathway^4^. Therefore, these animals contract scurvy unless they ingest AsA from food.

The expression of senescence marker protein-30 (SMP30), a lactone-hydrolyzing enzyme gluconolactonase and the penultimate enzyme in the AsA biosynthesis pathway^5^, has been shown to be decreased in the liver, kidney, and lung of aged mice^6^. SMP30-knockout (KO) mice are unable to synthesize AsA and are used as a closely related model animal to humans.

Skeletal muscle is a heterogeneous tissue containing thousands of fibers with different morphologies and functions^7^. Muscle fibers are classified into four groups, types I, IIa, IIb, and IId/x, in rodents according to characteristics such as myosin heavy chain (MHC) expression, contraction rate, ATPase activities, and myoglobin content^8–10^. Types I and IIa fibers work under oxidative conditions, exhibit higher resistance to fatigue and are mitochondria rich. Types IIb and IId/x fibers are mainly glycolytic, display lower resistance to fatigue and are mitochondria poor tissues^11^. ROS are continuously produced in skeletal muscles^12^, and the production of ROS is substantially increased by muscular exercise and promotes oxidation reactions that induce signaling and potentially damage biomolecules, such as proteins, lipids and DNA^1^. Recently, physical inactivity was also shown to induce ROS production in skeletal muscle and cause muscle atrophy^13,14^. Moreover, ROS levels are increased in subjects with aging-related sarcopenia and muscular diseases^15,16^. Additionally, exposure to hydrogen peroxide, a kind of ROS, promotes protein degradation in skeletal muscle via the ubiquitin-proteasome pathway^17,18^. The total amount of AsA in human skeletal muscle is approximately 40%^19^, and AsA protects the body from oxidative stress^20^. As shown in our previous study, plasma AsA concentrations in elderly women correlate well with their muscle strength and physical performance^21^. Additionally, in community-dwelling older people (Hertfordshire Cohort Study), higher AsA intake is only associated with physical performance (short chair rise time) in women^22^. These results prompted the hypothesis that AsA in muscle might act to maintain muscle weight and function through its function as an antioxidant by preventing excess oxidative states. However, the role of AsA in skeletal muscle remains unclear. Therefore, we examined whether AsA deficiency causes any defect in the structure and function of female mouse skeletal muscles using SMP30-KO mice. In this study, AsA deficiency caused muscle atrophy and significantly reduced muscle physical performance and the levels of ubiquitin ligase mRNAs. All these observed effects of AsA deficiency in muscles were restored by AsA supplementation.

## Results

### AsA and ROS levels in skeletal muscle

**Figure 1:**
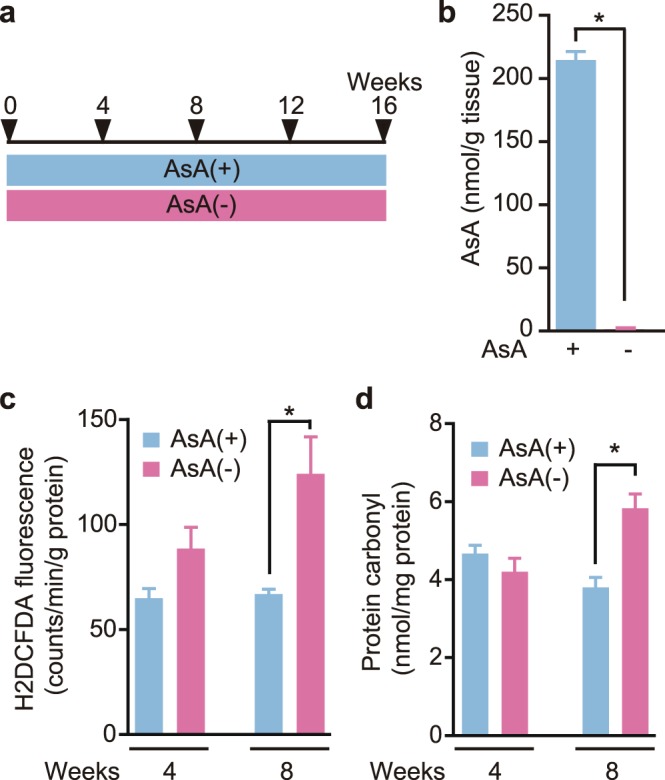
AsA, ROS, and protein carbonyl levels in skeletal muscle. (**a**) Schematic of AsA-sufficient (blue, AsA(+)) and -deficient (pink, AsA(−)) conditions during the experiment for 16 weeks. (**b**) Total AsA contents in the gastrocnemius muscles of the AsA(+) and AsA(−) groups at 4 weeks. n = 5 mice/group. (**c**) ROS levels in the soleus muscles of the AsA(+) and AsA(−) groups at 4 and 8 weeks. n = 4–5 mice/group. (**d**) Protein carbonyl levels in the soleus muscles of the AsA(+) and AsA(−) groups at 4 and 8 weeks. n = 4–5 mice/group. Values are presented as the mean ± SEM. *Significant difference at *p* < 0.05 compared with values for the AsA(+) group.

Eight-week-old female SMP30-KO mice were divided into two groups and housed with either free access to 1.5 g/L AsA water [AsA(+)] or AsA-free tap water [AsA(−)]and an AsA-free diet (Fig. 1a). The AsA content of skeletal muscle was evaluated at 4 weeks to clarify the effects on AsA deficiency on skeletal muscle. The AsA content in the gastrocnemius muscle from AsA(−) mice was 0.7% of the content in AsA(+) mice, indicating a significant difference between these two groups (Fig. 1b). Thus, AsA deficiency for 4 weeks was sufficient to deplete the AsA content in skeletal muscle.

AsA is known to scavenge ROS, such as hydroxyl radicals, singlet oxygen, and superoxide radicals, produced *in vivo*^1^. Therefore, we evaluated the ROS levels in the skeletal muscles of the AsA(+) and AsA(−) groups using 5-(and-6)-carboxy-2′,7′-dichlorodihydrofluorescein diacetate (H2DCFDA) as an indicator for ROS (Fig. 1c). A slightly higher ROS level was detected in the skeletal muscles of the AsA(−) group at 4 weeks, but no significant differences were observed between the AsA(+) and AsA(−) groups (Fig. 1c). Furthermore, at 8 weeks, ROS levels in the skeletal muscles from the AsA(−) group were significantly increased by 2-fold compared with those from the AsA(+) group (Fig. 1c). Next, we determined the protein carbonyl levels, which are stable oxidative stress markers (Fig. 1d). Protein carbonyl levels in the soleus did not change at 4 weeks between the AsA(+) and AsA(−) groups; however, at 8 weeks, protein carbonyl levels from the AsA(−) group were significantly (1.5-fold) higher than those from the AsA(+) group (Fig. 1d).

### Skeletal muscle weight and cross-sectional area (CSA) of muscle fibers

**Figure 2:**
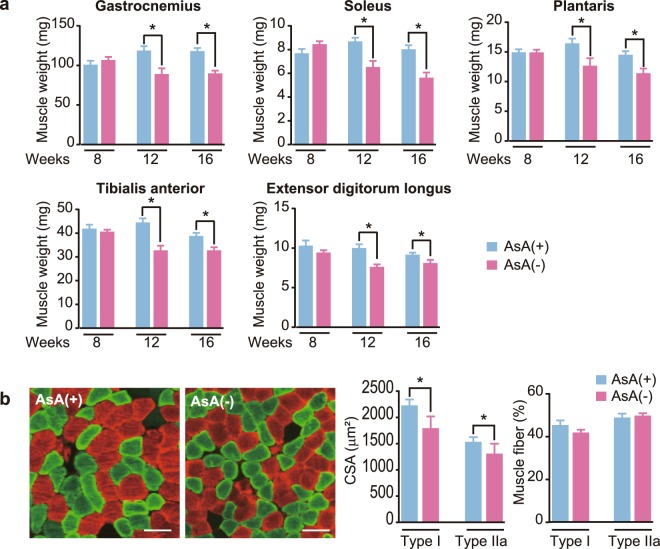
Loss of skeletal muscle weight and fiber atrophy during AsA deficiency. (**a**) Weights of the hindlimb skeletal muscles of the AsA(+) (blue) and AsA(−) (pink) groups at the indicated periods. n = 5–10 mice in the AsA(+) group and 5–10 mice in the AsA(−) group. (**b**) Panels: Images of cross-sections of the soleus muscles from the AsA(+) and AsA(−) groups at 16 weeks immunostained with muscle anti-MHC type I (red) and MHC type IIa (green) antibodies. Scale bar, 60 µm. Left graph: CSA of immunostained type I and type IIa fibers. n = 10 mice in both the AsA(+) and AsA(−) groups. Right graph: Ratio of the muscle fiber composition of soleus muscles from the AsA(+) and AsA(−) groups at 16 weeks. Numbers of immunostained types I and IIa fibers were counted and reported as a percentage relative to the total fibers. n = 10 mice in the AsA(+) group and 9 mice in the AsA(−) group. Values are presented as the mean ± SEM. *Significant difference at *p* < 0.05 compared with values for the AsA(+) group.

We first measured the weight of major hindlimb skeletal muscles, such as gastrocnemius, soleus, plantaris, tibialis anterior (TA), and extensor digitorum longus (EDL) muscles, at 8, 12, and 16 weeks to examine the effect of AsA deficiency on skeletal muscle (Fig. 2a). At 8 weeks, no significant differences were observed in the weights of all skeletal muscles examined between the AsA(+) and AsA(−) groups. However, the weights of the gastrocnemius, soleus, plantaris, TA, and EDL muscles in the AsA(−) group were significantly decreased to 75, 83, 77, 74, and 76%, respectively, at 12 weeks compared to those in the AsA(+) group (Fig. 2a). In addition, at 16 weeks, the weight of each muscle in the AsA(−) group was significantly decreased by 76, 70, 79, 85, and 88% compared with the values in the AsA(+) group, respectively. Based on these results, AsA deficiency might induce skeletal muscle atrophy.

Next, the CSA of each fiber, including types I and IIa, was measured to assess whether skeletal muscle weight loss occurred concomitantly with muscle fiber atrophy. Cross sections of the soleus muscles obtained from the AsA(+) and AsA(−) groups at 16 weeks were coimmunostained with two MHC-specific antibodies against type I (red) and type IIa (green) MHCs, as shown in Fig. 2b. The CSAs of both types I and IIa fibers from the AsA(−) group were significantly reduced by 28 and 25%, respectively, compared to the AsA(+) group (Fig. 2b). Furthermore, the ratio of types I and IIa muscle fibers from the AsA(−) group was measured to investigate the potential difference in the muscle fiber type composition. However, no differences were observed between these two groups (Fig. 2b).

### Physical performance

**Figure 3:**
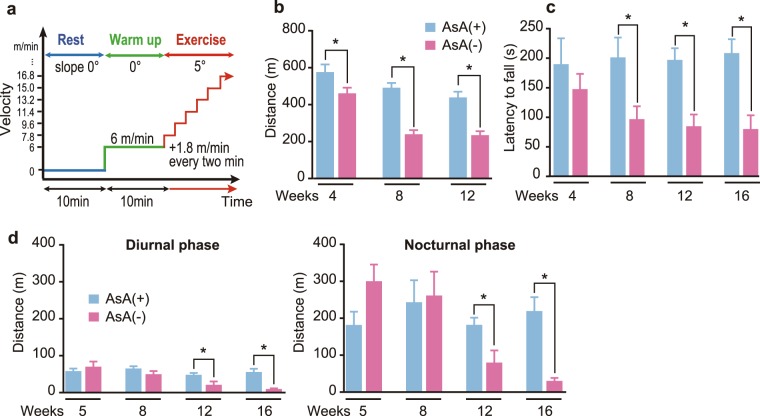
Effect of AsA deficiency on physical performance. (**a**) Schematic of the treadmill running paradigm. (**b**) Endurance performance was assessed by calculating the treadmill running distance. n = 9–10 mice in the AsA(+) group and 13–21 mice in the AsA(−) group. (**c**) Grip strength was measured using the wire hanging test and reported as the latency time when mice fell. n = 10–30 mice in the AsA(+) group and 15–33 mice in the AsA(−) group. (**d**) Home cage activity during the diurnal (left panel) and nocturnal phases (right panel). The distance each mouse traveled was recorded by three-point meters during each phase. n = 7 mice in the AsA(+) group and 5–8 mice in the AsA(−) group. Values are presented as the mean ± SEM. *Significant difference at *p* < 0.05 compared with values for the AsA(+) group.

AsA deficiency caused skeletal muscle weight loss and fiber atrophy, as shown above. Therefore, we assessed the physical performance of the AsA(−) and AsA(+) groups. Endurance performance was assessed using a treadmill, as shown in the scheme presented in Fig. 3a; grip strength was assessed using the wire hanging test, and home cage activity was examined using a three-point meter. Among these three physical performance tests, significant differences in the endurance performance were first observed between the two groups at 4 weeks (Fig. 3b). The endurance performance of the AsA(−) group at 4, 8, and 12 weeks was significantly decreased by 20, 50, and 46%, respectively, compared to that of the AsA(+) group (Fig. 3b). The grip strength of the AsA(−) group was significantly reduced by 52, 57, and 62% compared with that of the AsA(+) group at 8, 12, and 16 weeks, respectively (Fig. 3c). Home cage activity in both diurnal and nocturnal phases was not different between the two groups up to 8 weeks (Fig. 3d). However, home cage activity in the diurnal and nocturnal phases of the AsA(−) group was significantly decreased by 56 and 56% at 12 weeks and 83 and 86% at 16 weeks, respectively, compared with those of the AsA(+) group (Fig. 3d). Overall, at 12 weeks, significant differences in all measures of physical performance examined were observed between the AsA(+) and AsA(−) groups, although the timing was quite different among these three tests.

### Gene expression in skeletal muscle

**Figure 4:**
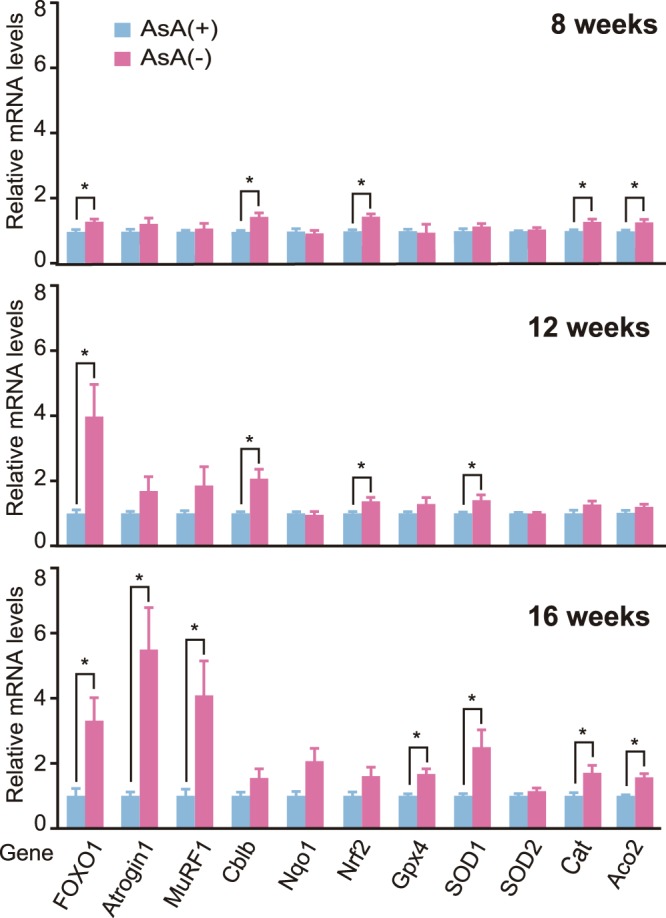
The mRNA expression levels of muscle atrophy- and oxidative stress-related genes during AsA deficiency. The mRNA expression levels of the indicated genes in TA muscles of the AsA(+) and AsA(−) mice were measured using qPCR at 8, 12, and 16 weeks. The mRNA expression levels were normalized to GAPDH mRNA and are shown as a ratio to the AsA(+) group. n = 5 mice in the AsA(+) group and 5–8 mice in the AsA(−) group. Values are presented as the mean ± SEM. *Significant difference at *p* < 0.05 compared with values for the AsA(+) group.

The expression levels of muscle atrophy-related genes, including forkhead box O1 (FOXO1), atrogin1/muscle atrophy F-box (MAFbx), muscle RING finger 1 (MuRF1), casitas B-lineage lymphoma proto-oncogene b (Cblb), and oxidative stress-related genes, including NAD(P)H dehydrogenase, quinone 1 (Nqo1), NF-E2-related factor 2 (Nrf2), glutathione peroxidase 4 (GPx4), superoxide dismutase 1 (SOD1), superoxide dismutase 2 (SOD2), catalase (Cat), and aconitase 2 (Aco2), in the skeletal muscle were examined using quantitative real-time polymerase chain reaction (qPCR) at 8, 12, and 16 weeks to examine the mechanism of skeletal muscle weight loss and fiber atrophy caused by AsA deficiency. At 8 weeks, levels of the FOXO1, Cblb, Nurf2, Cat and Aco2 mRNAs were significantly upregulated in the skeletal muscle of the AsA(−) group compared with those in the skeletal muscle of the AsA(+) group (Fig. 4). Furthermore, at 12 weeks, the most upregulated gene was FOXO1, followed by Cblb. At 16 weeks, the expression of FOXO1, atrogin1/MAFbx, and MuRF1 was significantly increased by 193, 418, and 268%, respectively, in the skeletal muscle of the AsA(−) group compared with that in the skeletal muscle of the AsA(+) group. In addition, GPx4, SOD1, catalase, and Aco2 genes were expressed at 89, 156, 60, and 52% higher levels, respectively, in the AsA(−) group than those in the AsA(+) group at 16 weeks (Fig. 4).

### Recovery of muscle atrophy and physical performance following AsA supplementation

**Figure 5:**
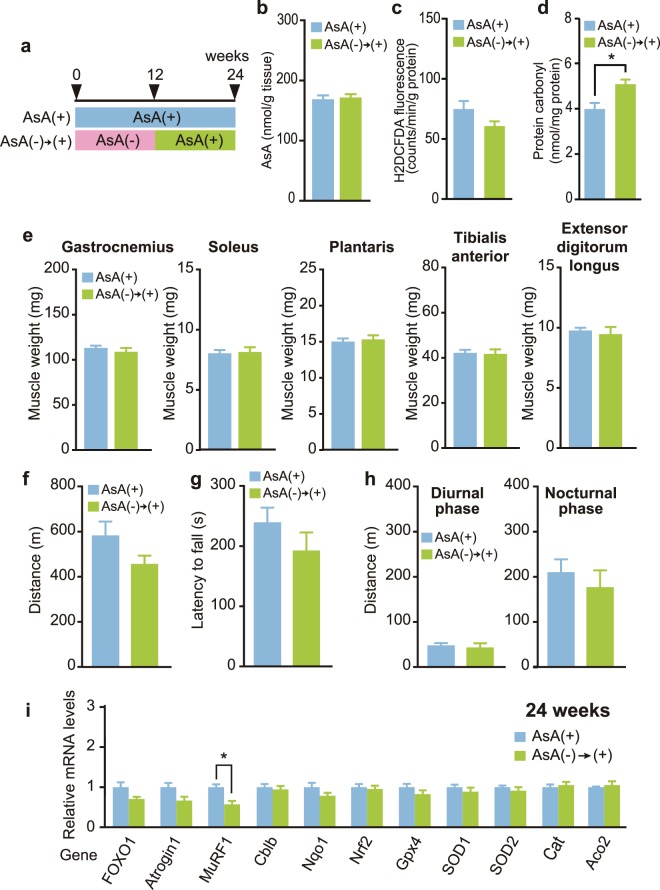
AsA supplementation reversed muscle atrophy and restored physical performance. (**a**) Schematic of AsA-sufficient [AsA(+)] and recovery [AsA(−) → (+)] conditions until 24 weeks. (**b**) Total AsA contents in the gastrocnemius muscles of the AsA(+) and AsA(−) → (+) groups at 24 weeks. n = 10 mice each in the AsA(+) and AsA(−) → (+) groups. (**c**) ROS levels in the soleus muscles of the AsA(+) and AsA(−) → (+) groups at 24 weeks. n = 5 mice in both the AsA(+) and AsA(−) → (+) groups. (**d**) Protein carbonyl levels in the soleus muscles of the AsA(+) and AsA(−) → (+) groups at 24 weeks. n = 5 mice in both the AsA(+) and AsA(−) → (+) groups. (**e**) Weights of the hindlimb skeletal muscles of the AsA(+) and AsA(−) → (+) groups at 24 weeks. n = 10 mice in both the AsA(+) and AsA(−) → (+) groups. (**f**–**h**) Effects of AsA supplementation on physical performance at 24 weeks. (**f**) Endurance performance was assessed using a treadmill. n = 7 mice in the AsA(+) group and n = 5 mice in the AsA(−) → (+) group. (**g**) Grip strength was measured using the wire hanging test. n = 17 mice in the AsA(+) group and n = 15 mice in the AsA(−) → (+) group. (**h**) Home cage activities during diurnal and nocturnal phases were measured using three-point meters. n = 15 mice in the AsA(+) group and n = 9 mice in the AsA(−) → (+) group. (**i**) Expression levels of muscle atrophy-, oxidative stress- and antioxidant-related genes at 24 weeks. Expression levels of the indicated genes in TA muscles of the AsA(+) and AsA(−) → (+) groups were measured using qPCR. The mRNA expression levels were normalized to GAPDH mRNA and are presented as a ratio to AsA(+) mice. Values are presented as the mean ± SEM. *Significant difference at *p* < 0.05 compared with values for the AsA(+) group.

As described above, muscle weight loss, muscle fiber atrophy, and decreased physical performance were observed in mice with AsA deficiency. AsA was administered to mice in the AsA(−) group beginning at 12 weeks, when all tested defects and muscle atrophy were observed, until 24 weeks to confirm that AsA deficiency caused these effects on skeletal muscle (Fig. 5a). Interestingly, the decrease in the AsA content, elevated ROS level, and skeletal muscle weight loss caused by AsA deficiency for 12 weeks were restored by AsA supplementation for 12 weeks (Fig. 5b,c,e); however, the protein carbonyl level in the AsA(−) → (+) group did not recover completely to the AsA(+) control level (Fig. 5d). Moreover, all tested physical performance metrics, including endurance performance, grip strength, and home cage activity, in the AsA(−) → (+) group was restored to approximately the same levels as those in the AsA(+) group (Fig. 5f–h). Furthermore, the expression levels of the examined genes, with the exception of MuRF1, were not different between the AsA(−) → (+) and AsA(+) groups (Fig. 5i).

## Discussion

In this study, AsA deficiency in skeletal muscles caused muscle atrophy concomitant with high expression of muscle atrophy-related genes. Furthermore, the physical performance evaluated using endurance performance, grip strength, and home cage activity tests decreased following AsA deficiency. Interestingly, these decreases in physical performance and increased expression levels of muscle atrophy-related genes were restored by AsA supplementation.

We previously reported that plasma AsA concentrations in elderly women correlate well to their muscle strength and physical performance^21^. In addition, in community-dwelling older people in the Hertfordshire Cohort Study, higher AsA intakes were associated with physical performance in only women^22^. Therefore, in this study, we used female mice and investigated the effect of AsA deficiency in mouse skeletal muscle.

The AsA contents in gastrocnemius muscles from the AsA(−) group were low at 4 weeks compared to the AsA(+) group, and a slightly higher ROS level was detected in the soleus muscles of the AsA(−) group at 4 weeks. Furthermore, noticeably higher ROS levels were observed in the soleus muscles of the AsA(−) group at 8 weeks than in the AsA(+) group. In addition, higher protein carbonyl levels were observed in the AsA(−) group at 8 weeks than in the AsA(+) group. ROS levels in muscles may be due to the activities of intracellular antioxidant enzymes, such as superoxide dismutase (SOD), glutathione peroxidase (GPx), and catalase, which might contribute to the reduced ROS levels. A continuous AsA deficiency resulted in higher ROS levels at 8 weeks. In fact, no differences in the expression levels of SOD1, SOD2, and GPx4 genes were observed at 8 weeks between these two groups, although the expression levels of SOD1, GPx4, and catalase genes were significantly higher in the AsA(−) group than those in the AsA(+) group at 16 weeks. Based on these results, we predicted that antioxidant enzyme activity may not be altered in muscle and led to ROS accumulation at 8 weeks. As shown in our previous report, ROS levels in the brain, heart, lung, testis, soleus muscle, plantaris muscle, stomach, small intestine, and large intestine were significantly increased by 27, 39, 29, 32, 81, 28, 43, 42, and 64%, respectively, in AsA(−) mice at 7 weeks compared with those in AA(+) mice^23^. Thus, AsA deficiency resulted in high ROS levels in various tissues.

The AsA(−) group at 4 weeks showed decreased endurance performance, which was the first significant physical performance deficit symptom observed in the AsA(−) group. Endurance performance was the exercise with the highest load among the three physical performance tests examined in this study. The oxygen consumption during exercise increased 10–15-fold compared to oxygen consumption during resting, and it increased by 100-fold in the active skeletal muscle^24^. Therefore, we postulated that the ROS level increased during exercise and decreased endurance performance due to oxidative stress in active skeletal muscle. Actually, the levels of oxidative stress markers, such as antioxidant enzymes and antioxidants, are increased in skeletal muscle after strenuous exercise^25^. However, the levels of oxidative stress markers do not increase after moderate exercise^26^. Thus, the balance is very important, and a high oxidative stress level occurs if the balance is disrupted. In the present study, the AsA(−) group ran a significantly shorter distance than the AsA(+) group without a loss in muscle weight, suggesting that the AsA-deficient group displayed a lower physical performance at 4 weeks. In addition, the grip strength of the AsA(−) group decreased at 8 weeks when the ROS amount was much higher compared with the AsA(+) group. Additionally, all of the tested physical performance measures decreased at 12 weeks. These results support the hypothesis that elevated oxidative damage correlates with a diminished functional capacity^27^.

ROS have been shown to trigger apoptosis in mouse skeletal muscle^28^ and cause muscle atrophy via the autophagy-lysosome pathway^14^. Moreover, increased ROS production and reduced antioxidant gene expression are related to muscle catabolism^29–32^. ROS activate proteasome-dependent protein degradation by inducing the expression of muscle-specific E3 ligases, such as MuRF1 and atrogin1/MAFbx^18^. Moreover, treatment of skeletal muscle cells with hydrogen peroxide induces Cblb gene expression^33^. Significantly higher Cblb and atrogin1/MAFbx expression was detected in skeletal muscle from AsA(−) mice at 12 and 16 weeks than in AsA(+) mice and thus might cause muscle atrophy. Moreover, exposure to ROS, particularly hydrogen peroxide, seems to promote protein degradation in skeletal muscle through the ubiquitin-proteasome pathway^18^. Thus, the present study confirmed high ROS levels in skeletal muscle and muscle atrophy in AsA(−) mice.

Interestingly, the decreases in muscle weight and physical performance caused by a 12-week AsA deficiency were clearly restored to the levels of AsA(+) controls by AsA oral supplementation. AsA supplementation for 12 weeks was sufficient to restore the AsA content in the muscle and may affect the redox signaling pathway to repress the upregulated expression of muscle atrophy-related genes via its antioxidant activity. By restoring muscle weight and redox state, normal physical performance might be regained.

In conclusion, longer-term AsA deficiency is associated with muscle wasting, that this can be reversed by restoring AsA levels, and that there may be some concurrent ROS production.

## Methods

### Animals

Animal experiments were conducted in accordance with the animal care and use protocol approved by the Institutional Animal Care and Use Committee of Tokyo Metropolitan Institute of Gerontology (TMIG) (Permit Number: 18033) and with the Guidelines for the Care and Use of Laboratory Animals of TMIG. SMP30-KO mice were generated using the gene targeting technique described previously^34^. Female SMP30^−/−^ mice were mated with male SMP30^Y/−^ mice to produce SMP30-KO mice. Female SMP30-KO mice were used in this study. After weaning at 4 weeks old, mice were administered 1.5 g/L AsA in drinking water for 8 weeks. At 8 weeks of age, these mice were divided into the following two groups: the AsA-sufficient [AsA(+)] group and the AsA-deficient [AsA(−)] group (Fig. 1a). The AsA(+) group had free access to water containing 1.5 g/L AsA and 10 µM ethylenediaminetetraacetic acid (EDTA), whereas the AsA(−) group had free access to tap (AsA-free) water. For the recovery experiment, AsA(−) mice were administered 1.5 g/L AsA for 12 weeks [AsA(−) → (+)] after having been administered AsA-free water for 12 weeks (Fig. 5a). As a control group, AsA(+) mice were administered 1.5 g/L AsA for 24 weeks. Water bottles were changed every three or four days during the experiment. All mice were fed an AsA-free diet (CL-2, CLEA Japan, Tokyo, Japan). At 4, 8, 12, and 16 weeks, the gastrocnemius, soleus, plantaris, TA, and EDL muscles were collected, weighed, frozen in liquid nitrogen and stored at −80 °C until use.

### Measurement of AsA contents

AsA contents were measured using a high-performance liquid chromatograph (HPLC) and electrochemical detector (Nihon Waters, Tokyo, Japan), as described previously^35^. The gastrocnemius muscle was homogenized in 14 volumes of 5.4% metaphosphoric acid (MPA, Wako Pure Chemical, Osaka, Japan) using a Minilys Tissue Homogenizer three times (Bertin Technologies, Montigny-le-Bretonneux, France) at the maximum speed for 30 sec and centrifuged at 21,000 × g for 15 min at 4 °C. Supernatants obtained after centrifugation were suitably diluted, and samples were treated with Tris (2-carboxyethyl) phosphine hydrochloride and then incubated for 2 h at 4 °C to reduce dehydroascorbic acid to AsA to measure the total AsA (AsA plus dehydroascorbic acid is an oxidized form of AsA) levels. Thereafter, 5% MPA was added to the samples, which were centrifuged at 21,000 × g for 10 min at 4 °C. Then, the resulting supernatants were analyzed by HPLC using an Atlantis dC18 5 µm column (4.6 × 150 mm, Nihon Waters). The mobile phase consisted of 50 mM phosphate buffer (pH 2.8), 54 μM EDTA and 2% methanol at a flow rate of 1.3 mL/min, and electrical signals were recorded using an electrochemical detector with a glassy carbon electrode at +0.6 V.

### ROS determination

ROS were measured using H2DCFDA (Thermo Fisher Scientific, Waltham, MA, USA) as described previously^23^. Soleus muscles were homogenized with 14 volumes of homogenization buffer consisting of 50 mM sodium phosphate buffer (pH 7.4), 0.5 mM phenylmethanesulfonyl fluoride, 1 mM EDTA, and cOmplete™ EDTA-free Protease Inhibitor Cocktail (F. Hoffmann-La Roche, Basel, Switzerland) using a hand-held homogenizer (Mojimojikun, Nippon Genetics, Tokyo, Japan), and the homogenate was centrifuged at 900 × g for 15 min at 4 °C. One hundred microliters of 50 μM H2DCFDA, 25 μL of supernatants containing 5 μg total protein, and 75 μL of homogenization buffer were mixed to yield a 200 μL total reaction volume. Changes in fluorescence intensity were measured every 5 min for 120 min at 37 °C using a 2104 EnVision™ Multilabel Reader (PerkinElmer, Waltham, MA, USA) with excitation and emission wavelengths set at 485 and 535 nm, respectively. The ROS level in muscle was calculated using values from 90 to 120 min and expressed as counts/min/g protein. The protein concentration was determined by a BCA protein assay kit (Thermo Fisher Scientific) using bovine serum albumin as a standard.

### Protein carbonyl

The protein carbonyl level was determined by the 2,4-dinitrophenylhydrazine (DNPH, Aldrich, St. Louis, MO, USA) method as described previously^36^. Soleus muscles were homogenized with 20 volumes of 50 mM Tris-HCl (pH 7.4) and 1 mM EDTA by using a hand-held homogenizer. The supernatant was obtained by centrifugation at 10,000 × g for 30 min at 4 °C. Proteins were precipitated with 10% trichloroacetic acid. The precipitates were treated with 250 μL either 2 N HCl containing 10 mM DNPH or 2 N HCl alone as a control for 1 h at 15 °C and then centrifuged at 10,000 × g for 10 min at 4 °C. The precipitates were washed three times with 500 μL ethanol with ethyl acetate (1:1) and dissolved in 100 μL of 8 M urea. Absorbance at 370 nm was measured, and carbonyl contents were expressed as nmol per mg protein, calculated using a molar extinction coefficient of 22000 M^−1^ L^−1^. The protein content of soleus muscle extracts was determined by a BCA protein assay kit using bovine serum albumin as a standard. The value was normalized for the protein content.

### Immunofluorescence and histochemical staining

Frozen sections of the soleus muscle (10 µm) were blocked with 5% normal goat serum in phosphate-buffered saline containing 0.1% Triton X-100 for 30 min at room temperature and incubated for 120 min at room temperature with anti-MHC I (1:300, dilution, BA-F8, Developmental Studies Hybridoma Bank, Iowa, USA) and anti-MHC IIa (1:300 dilution, SC-71, Developmental Studies Hybridoma Bank) antibodies. Sections were washed three times with phosphate-buffered saline for 5 min each and then incubated with Alexa Fluor 555-conjugated goat anti-mouse Ig2b (1:1,000 dilution, Invitrogen, Carlsbad, CA) for MHC I or Alexa Fluor 488-conjugated goat anti-mouse IgG1 (1:1,000 dilution, Invitrogen) for MHC IIa for 60 min at room temperature. Sections were washed and covered with Fluoromount/Plus (COSMO BIO CO., LTD., Tokyo, Japan). The fluorescence images were detected and captured with a FLUOVIEW FV10i laser scanning confocal microscope (OLYMPUS, Tokyo, Japan). The number of muscle fibers and cross-sectional areas were determined in 3 randomly selected square fields (840 × 840 µm) from each section using WinROOF image processing software (Mitani Corp., Tokyo, Japan).

### Behavioral tests

Mice were handled for 5 min for 3 consecutive days before undergoing the series of physical performance tests described below.

### Treadmill

Endurance performance was assessed using a treadmill, as shown in the scheme presented in Fig. 3a. A motorized treadmill (LE 8710MTS; Control Panlab Instrument, Barcelona, Spain) was used to measure indexes defining endurance performance, as previously described with minor modifications^37^. During the treadmill test, mice were forced to run on a motor-driven treadmill. Mice were placed on the apparatus for 10 min and allowed to acclimate to the treadmill. Mice were provided with a 10 min warm-up period at 6 m/min and a 0° inclination. After warming up, the angle was fixed at 5°, and the speed was incrementally increased by 1.8 m/min every 2 min until the mouse reached exhaustion. A 0.2 mA electric current was delivered when mice reached the shock grid at the rear of the treadmill to encourage the mice to run. Exhaustion was defined as spending more than 5 continuous sec on the shock grid.

### Grip strength test

A wire hanging chamber (WH-3002; O’HARA & CO., LTD., Tokyo, Japan) was used to measure forelimb and hindlimb grip strength, as described previously^38^. Mice were placed on the cage top and then inverted and suspended. The latency time when mice fell was recorded (cutoff = 300 sec). The test was repeated two times at a 30 min interval. The maximum latency time was used as the result.

### Home cage activity

Spontaneous home cage activity was continuously measured using a three-point meter, which measures the number of interruptions of the infrared beams positioned in the X and Y axes around the cage (O’HARA & CO., LTD.). Mice were housed individually in cages containing wood-chip bedding. A 12 h light-dark cycle (lights on at 8:00 A.M.) was used. Infrared sensors were located 35 mm above the floor of the home cage and detected the movement in the position of the whole body of the mouse; the distance the animal traveled was recorded every 1 h. After the mice were habituated to their new cage for 6 days, we recorded and calculated their activity on the seventh day during the diurnal and nocturnal phases.

### Extraction of RNA and cDNA Synthesis

Total RNA was extracted from the whole TA muscle from one hindlimb using ISOGEN (Wako Pure Chemical)^39^. RNA concentrations were determined and confirmed as free from protein contamination by measuring the absorbance at 260 and 280 nm. Then, cDNAs were synthesized using SuperScript III reverse transcriptase (Invitrogen) according to the manufacturer’s protocol. The cDNA was stored at 80 °C until use.

### qPCR

Using the THUNDERBIRD® qPCR Mix (Toyobo, Osaka, Japan), qPCR was performed according to the manufacturer’s protocol. The primers for FOXO1, atrogin1 (atrogin1/MAFbx), MuRF1, Cblb, Nqo1, Nrf2, GPx4, SOD1, SOD2, Cat, Aco2, and glyceraldehyde-3-phosphate dehydrogenase (GAPDH) were purchased from Integrated DNA Technologies, Inc. (Coralville, IA, USA) (Supplemental Table S1). The reactions were performed using a real-time PCR instrument (StepOne Plus, Applied Biosystems, Foster City, CA, USA). The amplification protocol consisted of denaturation at 95 °C for 1 min, followed by 40 cycles of 95 °C for 15 sec and 60 °C for 1 min. For the quantitative analysis of each mRNA expression level, a standard curve was designed; an aliquot of each experimental sample was used to generate standard curves. The relative expression levels of each gene were normalized to GAPDH. The normalized mRNA expression levels of each gene were evaluated, and the ratios to AsA(+) mice were determined.

### Statistical analysis

The results are presented as the mean ± standard errors of the mean (SEM). The probability of significant differences between experimental groups was determined with Welch’s t-test using GraphPad Prism 6 software (GraphPad Software Inc., San Diego, CA, USA). Differences were considered statistically significant at *p* < 0.05.

## Supplementary information

Supplementary_Table_S1

## References

[1] Winterbourn CC (2008). Reconciling the chemistry and biology of reactive oxygen species. Nat Chem Biol.

[2] Linster CL, Van Schaftingen E (2007). Vitamin C. Biosynthesis, recycling and degradation in mammals. FEBS J.

[3] Pohanka M (2012). Ascorbic acid: an old player with a broad impact on body physiology including oxidative stress suppression and immunomodulation: a review. Mini Rev Med Chem.

[4] Nishikimi M, Koshizaka T, Ozawa T, Yagi K (1988). Occurrence in humans and guinea pigs of the gene related to their missing enzyme L-gulono-gamma-lactone oxidase. Arch Biochem Biophys.

[5] Kondo Y (2006). Senescence marker protein 30 functions as gluconolactonase in L-ascorbic acid biosynthesis, and its knockout mice are prone to scurvy. Proc Natl Acad Sci USA.

[6] Maruyama N, Ishigami A, Kondo Y (2010). Pathophysiological significance of senescence marker protein-30. Geriatr Gerontol Int.

[7] Pette D, Staron RS (1990). Cellular and molecular diversities of mammalian skeletal muscle fibers. Rev Physiol Biochem Pharmacol.

[8] Spangenburg EE, Booth FW (2003). Molecular regulation of individual skeletal muscle fibre types. Acta Physiol Scand.

[9] Zierath JR, Hawley JA (2004). Skeletal muscle fiber type: influence on contractile and metabolic properties. PLoS biology.

[10] Haizlip KM, Harrison BC, Leinwand LA (2015). Sex-based differences in skeletal muscle kinetics and fiber-type composition. Physiology.

[11] Schiaffino S, Reggiani C (2011). Fiber Types in Mammalian Skeletal Muscles. Physiol Rev.

[12] Arbogast S, Reid MB (2004). Oxidant activity in skeletal muscle fibers is influenced by temperature, CO_2_ level, and muscle-derived nitric oxide. Am J Physiol Regul Integr Comp Physiol.

[13] Kondo H, Nakagaki I, Sasaki S, Hori S, Itokawa Y (1993). Mechanism of oxidative stress in skeletal muscle atrophied by immobilization. Am J Physiol Endocrinol Metab.

[14] Rodriguez-Hernandez A (2009). Coenzyme Q deficiency triggers mitochondria degradation by mitophagy. Autophagy.

[15] Siu PM, Pistilli EE, Alway SE (2008). Age-dependent increase in oxidative stress in gastrocnemius muscle with unloading. J Appl Physiol.

[16] Moylan JS, Reid MB (2007). Oxidative stress, chronic disease, and muscle wasting. Muscle & nerve.

[17] Li YP (2005). TNF-alpha acts via p38 MAPK to stimulate expression of the ubiquitin ligase atrogin1/MAFbx in skeletal muscle. FASEB J.

[18] Li YP, Chen Y, Li AS, Reid MB (2003). Hydrogen peroxide stimulates ubiquitin-conjugating activity and expression of genes for specific E2 and E3 proteins in skeletal muscle myotubes. Am J Physiol Cell Physiol.

[19] Peake JM (2003). Vitamin C: effects of exercise and requirements with training. Int J Sport Nutr Exerc Metab.

[20] Sen CK (2001). Antioxidants in exercise nutrition. Sports Med.

[21] Saito K (2012). A Significant Relationship between Plasma Vitamin C Concentration and Physical Performance among Japanese Elderly Women. J Gerontol A Biol Sci Med Sci.

[22] Martin H (2011). Does diet influence physical performance in community-dwelling older people? Findings from the Hertfordshire Cohort Study. Age and ageing.

[23] Kondo Y (2014). Potato chip intake increases ascorbic acid levels and decreases reactive oxygen species in SMP30/GNL knockout mouse tissues. J Agric Food Chem.

[24] Halliwell, B. & Gutteridge, J. M. C. *Free Radicals in Biology and Medicine*. (OUP Oxford, 2015). 10.1016/0748-5514(85)90028-5 3939136

[25] Dekkers JC, van Doornen LJ, Kemper HC (1996). The role of antioxidant vitamins and enzymes in the prevention of exercise-induced muscle damage. Sports Med.

[26] Lovlin R, Cottle W, Pyke I, Kavanagh M, Belcastro AN (1987). Are indices of free radical damage related to exercise intensity. Eur J Appl Physiol Occup Physiol.

[27] Howard C (2007). Oxidative protein damage is associated with poor grip strength among older women living in the community. J Appl Physiol.

[28] Adhihetty PJ, O’Leary MF, Chabi B, Wicks KL, Hood DA (2007). Effect of denervation on mitochondrially mediated apoptosis in skeletal muscle. J Appl Physiol.

[29] Barreiro E (2005). Both oxidative and nitrosative stress are associated with muscle wasting in tumour-bearing rats. FEBS letters.

[30] Arias-Diaz J (1997). Local production of oxygen free radicals and nitric oxide in rat diaphragm during sepsis: effects of pentoxifylline and somatostatin. Eur J Sur.

[31] Fagan, J. M., Ganguly, M., Tiao, G., Fischer, J. E. & Hasselgren, P. O. Sepsis increases oxidatively damaged proteins in skeletal muscle. *Arch Surg* **131**, 1326–1331; discussion 1331–1322, (1996). 10.1001/archsurg.1996.01430240080011 8956775

[32] Linke A (2005). Antioxidative effects of exercise training in patients with chronic heart failure: increase in radical scavenger enzyme activity in skeletal muscle. Circulation.

[33] Nikawa T (2004). Skeletal muscle gene expression in space-flown rats. FASEB J.

[34] Ishigami A (2002). Senescence Marker Protein-30 Knockout Mouse Liver Is Highly Susceptible to Tumor Necrosis Factor-α- and Fas-Mediated Apoptosis. Am J Pathol.

[35] Iwama M, Shimokado K, Maruyama N, Ishigami A (2011). Time course of vitamin C distribution and absorption after oral administration in SMP30/GNL knockout mice. Nutrition.

[36] Amano A (2013). Effects of Ascorbic Acid Deficiency on Protein and Lipid Oxidation in Livers from SMP30/GNL Knockout Mice. J Nutr Sci Vitaminol (Tokyo).

[37] Nishikawa M (2015). AST-120 ameliorates lowered exercise capacity and mitochondrial biogenesis in the skeletal muscle from mice with chronic kidney disease via reducing oxidative stress. Nephrol Dial Transplant.

[38] Kojima N (2008). Inducible cAMP early repressor acts as a negative regulator for kindling epileptogenesis and long-term fear memory. J Neurosci.

[39] Chomczynski P, Sacchi N (2006). The single-step method of RNA isolation by acid guanidinium thiocyanate-phenol-chloroform extraction: twenty-something years on. Nat Protoc.

