# Supplementary material for: Vitamin C deficiency causes muscle atrophy and a deterioration in physical performance
Source: Sci Rep. 2019 Mar 20;9:4702. doi: 10.1038/s41598-019-41229-7 (PMC6426981; doi:10.1038/s41598-019-41229-7)
Supplement: Supplementary file 1 — Supplementary_Table_S1 [file 41598_2019_41229_MOESM1_ESM.pdf]

Vitamin C deficiency causes muscle atrophy and a deterioration in physical performance

Shoko Takisawa [1,2,3,#], Tomoko Funakoshi [1,#], Tomofumi Yatsu [1,#], Kisaburo Nagata [3], Toshiro Aigaki [2], Shuichi Machida [4], Akihito Ishigami [1,*]

[1] Molecular Regulation of Aging, Tokyo Metropolitan Institute of Gerontology, Tokyo, 173-0015, Japan

[2] Cellular Genetics, Graduate School of Science and Engineering, Tokyo Metropolitan University, Tokyo, Japan 192-0397

[3] Department of Biomolecular Science, Faculty of Science, Toho University, Chiba, 274-8510, Japan

[4] Graduate School of Health and Sports Science, Juntendo University, Chiba, 270-1695 Japan

# These authors contributed equally to this work.

*** Corresponding author:** Akihito Ishigami, Ph.D., Molecular Regulation of Aging, Tokyo Metropolitan Institute of Gerontology (TMIG), 35-2 Sakae-cho, Itabashi-ku, Tokyo 173-0015, Japan. Phone +81-3-3964-3241, E-mail: ishigami@tmig.or.jp

Supplementary Table S1. Primer sets used for the qPCR analysis.

**Target gene**		**Sequence**
FOXO1	*Forward*	5'–CTGGGTGTCAGGCTAAGAGT–3'
	Reverse	5'–GGGGTGAAGGGCATCTTT–3'
Atrogin1/MAFbx	*Forward*	5'–AAGGCTGTTGGAGCTGATAGCA–3'
	Reverse	5'–CACCCACATGTTAATGTTGCC–3'
MuRF1	*Forward*	5'–TGACCACAGAGGGTAAAG–3'
	Reverse	5'–TGTCTCACTCATCTCCTTCTTC–3'
Cblb	*Forward*	5'–CACCCTTCTCCCAAGCATAA–3'
	Reverse	5'–AGACCGAACAGGAGCTTTGA–3'
Nqo1	*Forward*	5'–GGTAGCGGCTCCATGTACTC–3'
	Reverse	5'–AGACCTGGAAGCCACAGAAA–3'
Nrf2	*Forward*	5'–TTCTTTCAGCAGCATCCTCTCCAC–3'
	Reverse	5'–ACAGCCTTCAATAGTCCCGTCCAG–3'
GPx4	*Forward*	5'–CTCCATGCACGAATTCTCAG–3'
	Reverse	5'–TATCGGGCATGCAGATCG–3'
SOD1	*Forward*	5'–GGAACCATCCACTTCGAGCA–3'
	Reverse	5'–CCCATGCTGGCCTTCAGTTA–3'
SOD2	*Forward*	5'–GAACAATCTCAACGCCACCG–3'
	Reverse	5'–GCTGAAGAGCGACCTGAGTT–3'
Cat	*Forward*	5'–CAGATGAAGCAGTGGAAGGAG–3'
	Reverse	5'–AGGAATCCGCTCTCTGTCAA–3'
Aco2	*Forward*	5'–GTACCATGTGGCCTCTGTCC–3'
	Reverse	5'–GTTCAACCGTTTACGGACAAT–3'
GAPDH	*Forward*	5'–TGAAGCAGGCATCTGAGGG–3'
	Reverse	5'–CGAAGGTGGAAGAGTGGGAG–3'

FOXO1, forkhead box O1; Atrogin1/MAFbx, Atrogin1/muscle atrophy F-box; MuRF1, muscle RING finger 1; Cblb, casitas B-lineage lymphoma proto-oncogene b; Nqo1, NAD(P)H dehydrogenase, quinone 1; Nrf2, NF-E2-related factor 2; GPx4, glutathione peroxidase 4; SOD1, superoxide dismutase 1; SOD2, superoxide dismutase 2; Cat, catalase; Aco2, aconitase 2; GAPDH, glyceraldehyde-3-phosphate dehydrogenase
